# Seizures and Epilepsy in Patients With Untreated Cerebral Cavernous Malformations: A Prospective, Population-Based Cohort Study

**DOI:** 10.1212/WNL.0000000000214387

**Published:** 2025-11-13

**Authors:** Abel Clemens Adriaan Sandmann, William Peter Vandertop, Philip Michael White, Dagmar Verbaan, Jonathan M. Coutinho, Rustam Al-Shahi Salman

**Affiliations:** 1 Department of Neurology, Amsterdam UMC, University of Amsterdam, the Netherlands;; 2 Neurovascular Disorders, Amsterdam Neuroscience, the Netherlands;; 3 Department of Neurosurgery, Amsterdam UMC, University of Amsterdam, the Netherlands;; 4 Translational and Clinical Research Institute, Newcastle University, United Kingdom; and; 5 Centre for Clinical Brain Sciences, University of Edinburgh, United Kingdom.

## Abstract

**Background and Objectives:**

Treatment decisions for patients with epileptic seizures due to cerebral cavernous malformations (CCMs) are challenging because of a paucity of studies. We aimed to assess the rates and risk factors of CCM-related seizures during long-term follow-up without CCM intervention.

**Methods:**

This population-based study included people aged 16 years or older in Scotland who were newly diagnosed with CCM during 1999–2003 or 2006–2010, using brain MRI or pathology. From the initial presentation that led to CCM diagnosis, during prospective follow-up without CCM intervention, we analyzed the rates and risk factors for epileptic seizure(s) after any presentation, a first-ever epileptic seizure, seizure recurrence after a first unprovoked epileptic seizure, and 2-year/5-year seizure freedom among patients with CCM-related epilepsy (CRE).

**Results:**

We included 300 patients with CCM (median age 44 years, interquartile range [IQR] 32–57, 159 [53%] female). During a median follow-up of 15 years (IQR 8–20), 98 (33%) patients experienced epileptic seizure(s) after any presentation, which was associated with age (adjusted hazard ratio [aHR] 0.82, 95% CI 0.71–0.95, per 10-year increment, *p* = 0.008) and initial presentation with epileptic seizure(s) (aHR 12.94, 95% CI 7.11–23.52, *p* < 0.001). Of 189 patients who had never experienced epileptic seizures at initial presentation, 6% (95% CI 2%–9%) experienced a first-ever epileptic seizure within 10 years. For 64 patients with a first unprovoked epileptic seizure at initial presentation or during follow-up, the risk of seizure recurrence within 10 years was 80% (95% CI 70%–91%), which was lower with antiseizure medicine(s) therapy after the first seizure (aHR 0.36, 95% CI 0.19–0.70, *p* = 0.003). Among 110 patients who had CRE at initial presentation or developed CRE during follow-up, 82% (95% CI 74%–91%) became 2-year seizure-free and 62% (95% CI 51%–72%) 5-year seizure-free at 10 years, mostly while on antiseizure medicine monotherapy (44/75 [59%] and 40/60 [67%], respectively).

**Discussion:**

The risk of a first-ever epileptic seizure from CCM is low. Four of 5 patients who experience a first unprovoked epileptic seizure develop a recurrent seizure within 10 years, justifying diagnosis of CRE after a first unprovoked epileptic seizure due to CCM. Most of the patients with CRE achieve 2-year/5-year seizure freedom within 10 years. These long-term findings can inform patients and guide clinical practice.

## Introduction

Cerebral cavernous malformations (CCMs) are intracranial vascular malformations that may be detected incidentally, with a prevalence of 1 in 625 asymptomatic people.^1^ CCMs may also be diagnosed if they cause epileptic seizures or new focal neurologic deficits (FNDs), either of which may be provoked by a concomitant intracranial hemorrhage (ICH).^2^ Epileptic seizures due to CCM without radiologic evidence of new ICH result from hemosiderin deposition in the adjacent brain parenchyma through leaky junctions between endothelial cells.^3,4^ Blood products may induce local inflammation and gliosis, triggering epileptogenic activity, particularly in cortical regions or the temporal lobe.^5^

Risks of CCM-related seizures often disrupt personal and professional life,^6^ and may lead to a reduced quality of life, particularly due to impaired mental health.^7,8^ After a first epileptic seizure due to CCM, treatment with antiseizure medicine(s) (ASM) is recommended due to the high risk of developing a recurrent seizure.^3,9^ This may justify diagnosis of CCM-related epilepsy (CRE) after the first seizure, according to the International League Against Epilepsy (ILAE) recommendation to diagnose epilepsy if the risk of seizure recurrence after a first unprovoked epileptic seizure exceeds 60% within 10 years,^10^ although this risk has previously only been assessed over 5 years of follow-up for CCM.^9^ Diagnosis of CRE may further affect quality of life due to driving restrictions.^11^ Furthermore, some patients with CRE may develop medically intractable seizures despite ASM polytherapy.^9^ Such cases often prompt consideration of CCM interventions (e.g., pure lesionectomy, extended resection, laser interstitial thermal therapy, or stereotactic radiosurgery).^5^

Until a randomized controlled trial is conducted, treatment decisions for CCM must rely on indirectly weighing the up-front risks of CCM interventions against those of medical management alone. However, long-term risks and independent risk factors for seizure and epilepsy outcomes with medical management remain unclear. In addition, although short-term seizure freedom may be achieved with ASM treatment,^9^ its rate and determinants over the long term are unknown. Therefore, our objective was to report the final follow-up of a longstanding prospective, population-based, inception cohort study of patients with CCM to determine the rates and risk factors for epileptic seizure(s) after any presentation, a first-ever epileptic seizure, seizure recurrence after a first unprovoked epileptic seizure, and seizure freedom among patients with CRE during long-term follow-up without CCM intervention.

## Methods

### Patient Selection

In this prospective, population-based cohort study, patients with CCM were identified by the Scottish Audit of Intracranial Vascular Malformations (SAIVMs), a National Health Service audit previously described in detail.^12^ In short, SAIVMs included adults aged 16 years or older who were first diagnosed with an intracranial vascular malformation during 1999–2003 and 2006–2010 in Scotland, using brain MRI or pathology. People diagnosed in 2004 and 2005 were not included because these years fell outside the predefined recruitment periods. Patients were identified through multiple overlapping sources of case ascertainment, including a nationwide collaborative network of specialists in neurology, neurosurgery, stroke medicine, radiology, and pathology, as well as central registers of hospital discharge records and death certificates.^12^

### Standard Protocol Approvals, Registrations, and Patient Consents

The Multicenter Research Ethics Committee for Scotland (MREC/98/0/48) as well as the Fife and Forth Valley Research Ethics Committee (08/S0501/76) approved this study. Opt-out consent was applicable to the collection and analysis of routinely recorded clinical data without any changes to care, whereas opt-in written informed consent at enrolment was required for additional self-reported data from the annual postal questionnaires. All analyses were performed on anonymized extracts. This article adheres to the Strengthening the Reporting of Observational Studies in Epidemiology guidelines for observational studies.^13^

### Data Collection

The study's inception point was a patient's “initial presentation”, defined as the date of symptom onset or medical consultation (if asymptomatic) leading to a diagnosis of CCM. Each CCM diagnosis was verified by certified neuroradiologists using the clinical diagnostic brain images based on established criteria.^14^ We categorized CCM location as supratentorial, infratentorial, or supratentorial and infratentorial, and registered whether a brainstem CCM or temporal cortical/superficial CCM was present. CCM size was measured as the largest diameter in any plane. If there were multiple CCMs, we assessed the symptomatic CCM, determined by the clinical features (including seizure semiology) and findings on the diagnostic brain images. In asymptomatic or incidentally diagnosed patients, we assessed the largest CCM.

Initial presentations that were classified as epileptic seizure(s) may or may not have been provoked by a symptomatic ICH, as confirmed by appropriate brain imaging. The presentation was classified as symptomatic ICH (without epileptic seizures(s)) or nonhemorrhagic FND based on previously published criteria.^2^ Symptomatic ICH was considered symptomatic due to epileptic seizure(s) if they were witnessed and occurred within 24 hours of ICH onset. Epileptic seizure(s) were considered unprovoked if they occurred without evidence of symptomatic ICH on brain imaging or at least 7 days after a symptomatic ICH.^15^ The presentation was incidental in patients who had unrelated symptoms or were asymptomatic. Moreover, we classified whether epileptic seizure(s) were definitely or possibly attributable to the CCM. Epileptic seizure(s) were possibly related to CCM if semiology was anatomically consistent with the CCM, but another cause or an alternative origin was possible.

Hospital medical records were reviewed to retrospectively identify whether a patient had a history of epileptic seizure(s) definitely or possibly related to CCM before initial presentation. Prospective follow-up involved annual surveillance of general practitioners and hospital medical records. In addition, we used annual questionnaires to general practitioners and consenting participants with CCM to identify prospective clinical events and prescriptions of ASM. We recorded whether patients were treated with monotherapy, polytherapy, or no ASM, and assumed that a patient would remain on the same number of ASM until otherwise specified.

### Statistical Analysis

Prospective follow-up started from the study's inception point and was censored at the date of CCM intervention, death, or last available follow-up, whichever occurred first. We performed several Kaplan-Meier time-to-event analyses. Cox proportional hazards regression models were conducted if there were sufficient events to include multiple covariates, relaxing the rule of a minimum of 10 events per variable.^16^ We considered the following covariates for inclusion in the model: age at initial presentation, sex, mode of initial presentation (epileptic seizure(s), ICH/FND, or incidental), presence (vs absence) of temporal cortical/superficial CCM, largest CCM diameter, multiple (vs single) CCM, and ASM treatment (if feasible considering nonrandomized evaluation of treatment effects). We assessed whether proportional hazards assumptions were fulfilled using log-log curves.

We quantified the occurrence of epileptic seizure(s) during follow-up after any presentation in the entire cohort. In addition, we assessed the risk of a first-ever epileptic seizure among patients who had never experienced epileptic seizure(s) at initial presentation, meaning they did not present with epileptic seizure(s) and had also not experienced epileptic seizure(s) before presentation. This analysis started at presentation for all patients. We primarily analyzed epileptic seizure(s) that were definitely or possibly related to CCM and performed sensitivity analysis to determine whether restriction to events that were only definitely related to CCM affected our analyses. The risk of a first-ever epileptic seizure was also assessed in a restricted analysis of patients with supratentorial CCM.

To evaluate the ILAE recommendation to diagnose epilepsy if the risk of seizure recurrence after a first unprovoked epileptic seizure exceeds 60% within 10 years for patients with CCM, we assessed the risk of seizure recurrence among patients who experienced a first unprovoked epileptic seizure during the study period, either at initial presentation (analysis started at presentation) or during follow-up (analysis started at the first unprovoked seizure). Patients who had experienced epileptic seizure(s) before initial presentation were excluded. We primarily analyzed recurrence of epileptic seizure(s) that were definitely or possibly related to CCM and performed sensitivity analysis of epileptic seizure(s) that were only definitely related to CCM.

Rates of 2-year and 5-year seizure freedom were analyzed among patients who met the definition of CRE because they experienced unprovoked epileptic seizure(s) due to CCM during the study period, either at initial presentation (analysis started at presentation) or during follow-up (analysis started at the CRE diagnosis), regardless of their prepresentation history of epileptic seizure(s). We primarily analyzed definite or probable CRE (i.e., unprovoked epileptic seizure(s) definitely or possibly related to CCM, respectively) and performed sensitivity analysis of only definite CRE.^3^ Analyses were performed using IBM SPSS Statistics version 28^17^ and MedCalc Statistical Software version 22.^18^

### Data Availability

Anonymized data supporting the findings of this study will be made available for scientific research on reasonable request to the corresponding author.

## Results

### Inception and Patient Selection

From 1999 to 2003 and 2006 to 2010, a first-ever diagnosis of at least 1 CCM was made in 306 adult residents in Scotland (295 on brain MRI, 6 at autopsy, and 5 after surgical resection). Six cases who were discovered incidentally during autopsy were excluded because they did not contribute to the outcome analyses. Among 300 included patients, the median age was 44 years (interquartile range [IQR] 32–57), 159 (53%) were women, and 55 (18%) had a temporal cortical/superficial CCM (Table 1).

**Table 1:** Baseline Characteristics

Variable	All patients (n = 300)
Age, y	44 (32–57)
Sex, female	159 (53)
Epileptic seizure(s) before initial presentation	52 (17)
Definitely related to CCM	46 (15)
Possibly related to CCM	6 (2)
Initial presentation with epileptic seizure(s)	100 (33)
Definitely related to CCM	95 (32)
Provoked by symptomatic ICH	12 (4)
Unprovoked by symptomatic ICH	83 (28)
Possibly related to CCM	5 (2)
Initial presentation without epileptic seizure(s)	200 (67)
Symptomatic ICH	38 (13)
FND	31 (10)
Incidental	131 (44)
Multiple CCMs	57 (19)
Location of CCM	
Supratentorial	215 (72)
Infratentorial	54 (18)
Supratentorial and infratentorial	31 (10)
Brainstem CCM	48 (16)
Temporal cortical/superficial CCM	55 (18)
Largest CCM diameter, mm	12 (8–18)
Associated DVA	28 (9)

Abbreviations: CCM = cerebral cavernous malformation; DVA = developmental venous anomaly; FND = nonhemorrhagic focal neurologic deficit; ICH = intracranial hemorrhage; IQR = interquartile range.

Data are median (IQR) or n (%).

**Figure 1:**
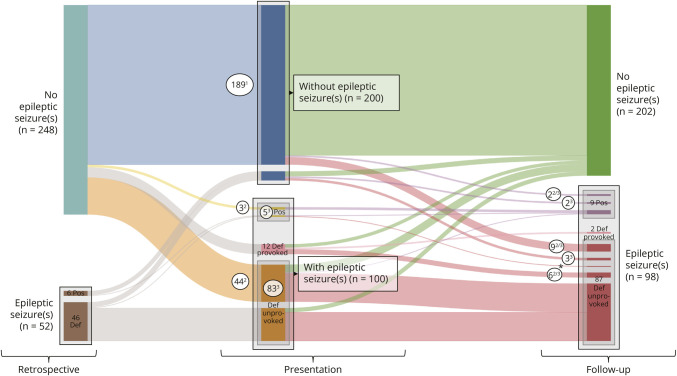
Sankey Diagram for the Course of Events of Epileptic Seizures Definitely or Possibly Related to CCM Before Initial Presentation (Retrospective, Left), at Initial Presentation (Middle), and During Follow-up (Right), as Well as the Patient Selection Per Outcome Analysis (1–3, Below) CCM = cerebral cavernous malformation; CRE = CCM-related epilepsy; def = definitely related to CCM; ICH = intracranial hemorrhage; pos = possibly related to CCM. ^1^Analysis 1: 189 patients did not present with epileptic seizure(s) and had also not experienced epileptic seizure(s) before initial presentation (blue flow), for whom the risk of a first-ever epileptic seizure was assessed. ^2^Analysis 2: 64 patients experienced a first unprovoked epileptic seizure definitely or possibly related to CCM at initial presentation (n = 47, of whom n = 44 definitely related to CCM [orange flow] and n = 3 possibly related to CCM [yellow flow]) or during follow-up (n = 17, of whom n = 15 definitely related to CCM [n = 9 after incidental presentation and n = 6 more than 7 days after initial presentation with epileptic seizure(s) definitely related to CCM provoked by symptomatic ICH] and n = 2 possibly related to CCM [top purple flow]), for whom the risk of seizure recurrence was assessed. ^3^Analysis 3: 110 patients met the definition of CRE, as they experienced unprovoked epileptic seizure(s) due to CCM at initial presentation (n = 88, of whom n = 83 definitely related to CCM and n = 5 possibly related to CCM) or during follow-up (n = 22, of whom n = 18 definitely related to CCM [top 2 and fourth red flows] and n = 4 possibly related to CCM [top 2 purple flows]), for whom the rates of 2-year and 5-year seizure freedom were assessed; *(third red flow) is included in sensitivity analysis of only definite CRE (n = 102), as epileptic seizure(s) definitely related to CCM occurred during follow-up after initial presentation with epileptic seizure(s) possibly related to CCM.

Figure 1 illustrates the course of epileptic seizures before initial presentation, at presentation, and during follow-up, as well as how patients were selected for each outcome analysis. Before initial presentation, 52 (17%) patients had experienced epileptic seizure(s) (n = 46 definitely related to CCM, n = 6 possibly related to CCM). Initial presentation with epileptic seizure(s) occurred in 100 (33%) patients (n = 95 definitely related to CCM, n = 5 possibly related to CCM). Of the 95 initial presentations with epileptic seizure(s) definitely related to CCM, 12 (13%) were provoked by a symptomatic ICH.

### Epileptic Seizure(s) After Any Presentation

During a median follow-up of 15 years (IQR 8–20), 98 (33%) patients experienced epileptic seizure(s) after any presentation (n = 89 definitely related to CCM, n = 9 possibly related to CCM). This outcome was associated with several variables in univariable analyses (Table 2). However, the only covariates that remained associated in multivariable analysis were age (adjusted hazard ratio [HR] 0.82, 95% CI 0.71–0.95, per 10-year increment, *p* = 0.008) and initial presentation with epileptic seizure(s) (vs incidental, adjusted HR 12.94, 95% CI 7.11–23.52, *p* < 0.001). These findings persisted in sensitivity analysis of events that were only definitely related to CCM (eTable 1).

**Table 2:** Cox Proportional Hazards Regression Model for Associations With the Occurrence of Epileptic Seizure(s) Definitely or Possibly Related to CCM During Follow-Up After Any Presentation

Covariate	All patients (n = 300)	Unadjusted HR (95% CI)	*p* Value	Adjusted HR (95% CI)	*p* Value
Age (10-y increments)	44 (32–57)	0.67 (0.58–0.78)	<0.001^a^	0.82 (0.71–0.95)	0.008^a^
Female (vs male) sex	159 (53)	0.58 (0.39–0.86)	0.007^a^	0.80 (0.53–1.22)	0.30
Mode of initial presentation					
Epileptic seizure(s)^b^ vs incidental	100 (33) vs 131 (44)	15.77 (9.01–27.61)	<0.001^a^	12.94 (7.11–23.52)	<0.001^a^
ICH/FND^c^ vs incidental	69 (23) vs 131 (44)	0.13 (0.02–1.01)	0.05	0.13 (0.02–1.00)	0.05
Presence (vs absence) of temporal cortical/superficial CCM	55 (18)	3.45 (2.29–5.20)	<0.001^a^	1.16 (0.76–1.79)	0.49
Largest CCM diameter (1 mm increments)	12 (8–18)	1.03 (1.01–1.06)	0.019^a^	1.00 (0.96–1.03)	0.83
Multiple (vs single) CCM	57 (19)	1.78 (1.14–2.79)	0.012^a^	1.49 (0.93–2.40)	0.10

Abbreviations: CCM = cerebral cavernous malformation; CI = confidence interval; FND = nonhemorrhagic focal neurologic deficit; HR = hazard ratio; ICH = symptomatic intracranial hemorrhage; IQR = interquartile range.

Data are median (IQR), n (%), or HR (95% CI).

a *p* Values were considered statistically significant (*p* < 0.05).

b Epileptic seizure(s) with or without concomitant ICH.

c Symptomatic ICH without epileptic seizure(s).

### First-Ever Epileptic Seizure

Among 200 patients who did not experience epileptic seizure(s) at initial presentation, 189 did not have a history of epileptic seizure(s) before presentation (Table 3). Baseline characteristics of these patients are provided in eTable 2; 135 of them had supratentorial CCM.

**Table 3:** Rates of a First-Ever Epileptic Seizure, Seizure Recurrence, and Seizure Freedom

Patients who had never experienced epileptic seizure(s) at initial presentation (n = 189)		
First-ever epileptic seizure	Definitely or possibly related to CCM	Definitely related to CCM
Proportion	11 (6)	9 (5)
Cumulative 5-y rate	3% (0%–6%)	2% (0%–5%)
Cumulative 10-y rate	6% (2%–9%)	4% (1%–8%)

Patients with a first unprovoked epileptic seizure at initial presentation or during follow-up		
Seizure recurrence	Definitely or possibly related to CCM (n = 64)	Definitely related to CCM (n = 59)
Proportion	49 (77)	43 (73)
Cumulative 1-y rate	64% (52%–76%)	62% (49%–75%)
Cumulative 5-y rate	78% (68%–89%)	75% (63%–87%)
Cumulative 10-y rate	80% (70%–91%)	77% (65%–89%)

Patients who had CRE at initial presentation or developed CRE during follow-up		
2-y seizure freedom	Definite or probable CRE (n = 110)	Definite CRE (n = 102)
Proportion	75 (68)	69 (68)
No ASM	12/75 (16)	9/69 (13)
1 ASM	44/75 (59)	44/69 (64)
2 or more ASM	19/75 (25)	16/69 (23)
Cumulative 5-y rate	62% (51%–72%)	65% (54%–76%)
Cumulative 10-y rate	82% (74%–91%)	84% (75%–92%)

5-y seizure freedom	Definite or probable CRE (n = 110)	Definite CRE (n = 102)
Proportion	60 (55)	56 (55)
No ASM	8/60 (13)	7/56 (13)
1 ASM	40/60 (67)	39/56 (70)
2 or more ASM	12/60 (20)	10/56 (18)
Cumulative 5-y rate	23% (14%–32%)	24% (15%–34%)
Cumulative 10-y rate	62% (51%–72%)	65% (54%–76%)

Abbreviations: ASM = antiseizure medicine(s); CCM = cerebral cavernous malformation; CI = confidence interval; CRE = CCM-related epilepsy; IQR = interquartile range.

Data are median (IQR), n (%), or rate (95% CI).

**Figure 2:**
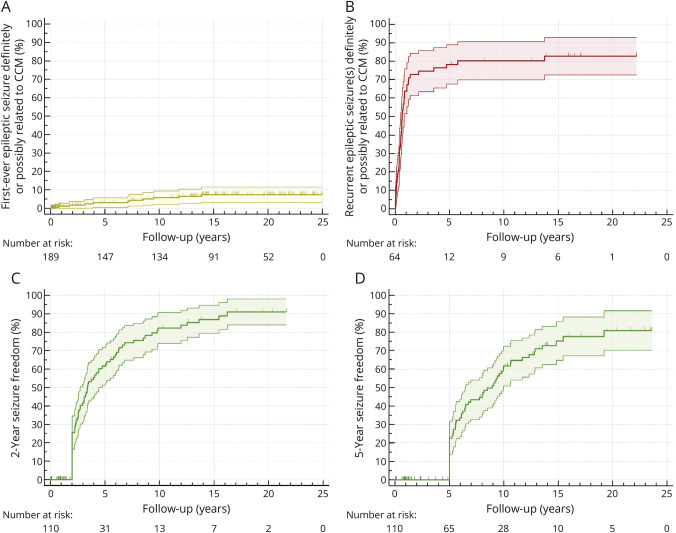
Kaplan-Meier Analyses for the Progression to a First-Ever Epileptic Seizure, Seizure Recurrence, and Seizure Freedom (A) First-ever epileptic seizure definitely or possibly related to CCM among 189 patients who had never experienced epileptic seizure(s) at initial presentation. (B) Recurrent epileptic seizure(s) definitely or possibly related to CCM among 64 patients with a first unprovoked epileptic seizure definitely or possibly related to CCM at initial presentation or during follow-up. (C, D) 2-year (C) and 5-year (D) seizure freedom among 110 patients who had definite or probable CRE at initial presentation or developed definite or probable CRE during follow-up. CCM = cerebral cavernous malformation; CRE = CCM-related epilepsy.

During 2,675 person-years of follow-up (median 15 years, IQR 10–21), 11 (6%) patients experienced a first-ever epileptic seizure definitely or possibly related to CCM (10-year rate 6%, 95% CI 2%–9%, Figure 2A), who all had supratentorial CCM (11/135 [8%], 10-year rate 8%, 95% CI 3%–13%, eFigure 1A). The mode of initial presentation of these patients was incidental (n = 10) or symptomatic ICH (n = 1). Similar results were found in sensitivity analysis of 9 events that were only definitely related to CCM (eFigure 1B). There were insufficient events to investigate risk factors in multivariable analyses.

### Risk of Seizure Recurrence

Sixty-four patients experienced a first unprovoked epileptic seizure definitely or possibly related to CCM during the study period (Table 3), of whom 47 were at initial presentation (n = 44 definitely related to CCM, n = 3 possibly related to CCM) and 17 during follow-up (n = 15 definitely related to CCM, n = 2 possibly related to CCM; a median of 3 years [IQR 1–8] after initial presentation), all of whom had supratentorial CCM (eTable 2). After the first seizure, 30 (47%) patients were treated with ASM.

During 747 person-years of follow-up beyond the first unprovoked epileptic seizure (median 14 years, IQR 3–17), 49 (77%) patients experienced recurrent epileptic seizure(s) definitely or possibly related to CCM (all of which were unprovoked) after a median of 6 months (IQR 3–10). The risk of seizure recurrence within 10 years was 80% (95% CI 70%–91%, Figure 2B), which justifies diagnosing CRE after a first unprovoked epileptic seizure due to CCM. Recurrence of epileptic seizure(s) definitely or possibly related to CCM was associated with multiple CCMs (unadjusted HR 2.43, 95% CI 1.25–4.74, *p* = 0.009) and ASM treatment after the first seizure (unadjusted HR 0.43, 95% CI 0.24–0.77, *p* = 0.004) in univariable analyses, but in multivariable analysis, only ASM treatment after the first seizure remained associated (adjusted HR 0.36, 95% CI 0.19–0.70, *p* = 0.003, Table 4).

**Table 4:** Cox Proportional Hazards Regression Model for Associations With Recurrence of Epileptic Seizure(s) Definitely or Possibly Related to CCM Among Patients With a First Unprovoked Epileptic Seizure Definitely or Possibly Related to CCM at Initial Presentation or During Follow-Up

Covariate	All patients (n = 64)	Unadjusted HR (95% CI)	*p* Value	Adjusted HR (95% CI)	*p* Value
Age (10-y increments)^a^	34 (23–45)	0.83 (0.68–1.03)	0.09	0.92 (0.73–1.14)	0.44
Female (vs male) sex	25 (39)	0.66 (0.36–1.19)	0.17	0.77 (0.40–1.48)	0.43
Presence (vs absence) of temporal cortical/superficial CCM	17 (27)	1.34 (0.74–2.44)	0.33	1.52 (0.76–3.04)	0.24
Largest CCM diameter (1 mm increments)	12 (8–20)	1.03 (0.99–1.08)	0.13	1.04 (1.00–1.08)	0.07
Multiple (vs single) CCM	14 (22)	2.43 (1.25–4.74)	0.009^b^	1.78 (0.82–3.87)	0.15
Treatment with ASM after the first seizure	30 (47)	0.43 (0.24–0.77)	0.004^b^	0.36 (0.19–0.70)	0.003^b^

Abbreviations: ASM = antiseizure medicine(s); CCM = cerebral cavernous malformation; CI = confidence interval; HR = hazard ratio; IQR = interquartile range.

Data are median (IQR), n (%), or HR (95% CI).

a Age at initial presentation.

b *p* Values were considered statistically significant (*p* < 0.05).

In sensitivity analysis of 59 patients who experienced a first unprovoked epileptic seizure definitely related to CCM during the study period (n = 44 at initial presentation, n = 15 during follow-up), during 665 person-years of follow-up beyond the first unprovoked epileptic seizure (median 13 years, IQR 2–17), 43 (73%) patients experienced recurrent (unprovoked) epileptic seizure(s) definitely related to CCM after a median of 6 months (IQR 3–9, 10-year rate 77%, 95% CI 65%–89%, eFigure 2). In Cox regression sensitivity analysis of recurrence of epileptic seizure(s) definitely related to CCM, the same covariates were associated (eTable 3).

### Two-Year and 5-Year Seizure Freedom

The definition of definite or probable CRE was met by 110 patients during the study period (Table 3), of whom 88 experienced unprovoked epileptic seizure(s) definitely or possibly related to CCM at initial presentation (n = 83 definitely related to CCM, n = 5 possibly related to CCM) and 22 during follow-up (n = 18 definitely related to CCM, n = 4 possibly related to CCM; a median of 2 years [IQR 1–7] after initial presentation), all of whom had supratentorial CCM (eTable 2).

Following the definite or probable CRE diagnosis, there were 1,401 person-years of follow-up (median 14 years, IQR 5–18). Twenty patients had less than 2 years of follow-up and were censored, after which 75 (68%) patients achieved 2-year seizure freedom (10-year rate 82%, 95% CI 74%–91%, Figure 2C), and 26 patients had less than 5 years of follow-up, after which 60 (55%) patients achieved 5-year seizure freedom (10-year rate 62%, 95% CI 51%–72%, Figure 2D). Most of the patients who became 2-year and 5-year seizure-free were on ASM monotherapy (44/75 [59%] and 40/60 [67%], respectively). There were no covariates significantly associated with the occurrence of 2-year seizure freedom (Table 5). We were unable to include ASM treatment as covariate due to the high risk of confounding.

**Table 5:** Cox Proportional Hazards Regression Model for Associations With the Occurrence of 2-Year Seizure Freedom Among Patients Who Had Definite or Probable CRE at Initial Presentation or Developed Definite or Probable CRE During Follow-Up

Covariate	All patients (n = 110)	Unadjusted HR (95% CI)	*p* Value	Adjusted HR (95% CI)	*p* Value
Age (10-y increments)^a^	37 (27–51)	0.99 (0.85–1.16)	0.92	0.97 (0.82–1.15)	0.70
Female (vs male) sex	47 (43)	1.03 (0.66–1.63)	0.89	1.02 (0.64–1.64)	0.93
Presence (vs absence) of temporal cortical/superficial CCM	40 (36)	0.82 (0.51–1.30)	0.40	0.87 (0.53–1.41)	0.56
Largest CCM diameter (1 mm increments)	12 (9–18)	1.01 (0.97–1.04)	0.77	1.01 (0.98–1.04)	0.61
Multiple (vs single) CCM	26 (24)	0.75 (0.43–1.30)	0.30	0.72 (0.39–1.35)	0.31

Abbreviations: ASM = antiseizure medicine(s); CCM = cerebral cavernous malformation; CI = confidence interval; CRE = CCM-related epilepsy; HR = hazard ratio; IQR = interquartile range.

Data are median (IQR), n (%), or HR (95% CI). ASM treatment was not included as covariate due to the high risk of confounding.

a Age at initial presentation.

In sensitivity analysis of 102 patients who met the definition of definite CRE during the study period (n = 83 at initial presentation, n = 19 during follow-up), rates of 2-year and 5-year seizure freedom were similar (10-year rates 84%, 95% CI 75%–92%, eFigure 3A, and 65%, 95% CI 54%–76%, eFigure 3B, respectively). The Cox regression sensitivity analysis for the occurrence of 2-year seizure freedom also showed no significant associations with the included covariates (eTable 4).

## Discussion

In this prospective, population-based cohort study, during long-term untreated follow-up, we found that one-third of all patients with CCM experienced epileptic seizure(s) after any presentation, with younger age and initial presentation with epileptic seizure(s) being risk factors for this outcome. The risk of a first-ever epileptic seizure was low. The risk of seizure recurrence following a first unprovoked epileptic seizure was 80% within 10 years and was lower when ASM treatment was started after the first seizure. Most of the patients who met the definition of CRE achieved 2-year and 5-year seizure freedom, mostly while using ASM monotherapy.

In clinical practice, patients who experience epileptic seizures due to CCM and their clinicians aim to minimize the risk of further seizures and achieve seizure freedom, with current best estimates available over 5 years after diagnosis. The 5-year risk of seizure recurrence after a first unprovoked epileptic seizure has been reported to be 94% (95% CI 84%–100%),^9^ but the rates in the long term have been uncertain. In this study, we found a 10-year recurrence rate of 80% (95% CI 70%–91%), despite approximately half of patients being treated with ASM after their first seizure. The other half of patients were not treated with ASM likely because they were not diagnosed with epilepsy at the time. Since the risk of seizure recurrence following a first unprovoked epileptic seizure due to CCM exceeded 60% within 10 years, diagnosis of CRE is justified after the first unprovoked epileptic seizure, in accordance with the ILAE definition of epilepsy.^10^ The recurrence risk was highest in the initial year after the first unprovoked epileptic seizure (1-year rate 64%, 95% CI 52%–76%) and was elevated for patients who did not receive ASM therapy, supporting ILAE guidelines to prescribe ASM after a first unprovoked CCM-related seizure.^3^

Seizure freedom rates in patients with CRE have been reported in previous studies, but most provided short-term outcomes. In one study, 2-year seizure freedom was achieved by 47% (95% CI 27%–67%) at 5 years,^9^ and in another study by 65% of patients who were on ASM monotherapy during a median follow-up of 7 years.^19^ We provide 2-year and 5-year seizure freedom rates over 10 years of 82% (95% CI 74%–91%) and 62% (95% CI 51%–72%), respectively. These data demonstrate that long-term seizure control may be achieved under medical management without CCM intervention. Furthermore, although several switches of ASM type may have occurred, patients mostly received ASM monotherapy when reaching these outcomes. This may indicate that the likelihood of becoming seizure-free decreases when multiple ASMs are required, in which case medical management may not be sufficient.

Select patients with CRE who do not become seizure-free and meet the definition for drug-resistant epilepsy^20^ might benefit from surgical treatment. This is supported by a decision analysis of the treatment of symptomatic CCMs that found intervention to be superior to conservative management for patients who presented with CRE.^21^ In addition, rates of seizure freedom have been shown to be lower in patients with CRE than in those with cryptogenic epilepsy.^22^ In this study, the proportion of patients meeting the definition of CRE who did not achieve 2-year and 5-year seizure freedom was 32% and 45%, respectively. However, because of the observational study design, 18% and 24% of patients, respectively, were censored because of CCM intervention before the seizure freedom outcomes could be reached. Therefore, a randomized controlled trial remains warranted to compare surgical treatment and medical management for patients with CRE.

Patients with CCM who have never experienced epileptic seizures have a low risk of developing a first-ever epileptic seizure, as reflected by the small number of such events in our study, which precluded multivariable analysis. As mentioned previously,^9^ the actual risk may be even lower because patients with incidental CCM were likely underrepresented given the prevalence of CCM in 1 in 625 asymptomatic people.^1^ These findings suggest that prophylactic ASM treatment is not indicated if patients present without epileptic seizures.^6,9^ This likely also applies in the case of symptomatic ICH without epileptic seizures because there is no evidence supporting the benefit of prophylactic ASM following hemorrhagic stroke.^23^ This aligns with our finding that initial presentation with ICH/FND was not associated with epileptic seizure(s) during follow-up after any presentation.

Age may serve as a prognostic factor for seizure and epilepsy outcomes from CCM. Younger patients had a higher risk of experiencing epileptic seizure(s) during follow-up after any presentation, potentially because highly epileptogenic CCMs become symptomatic early in life.^5^ This may suggest greater seizure burden for younger patients and/or better seizure-control in older patients.^24^ Given that younger patients face long-term cumulative risks, extrapolating these risks to their lifetimes may exceed the immediate risks associated with surgical intervention. Therefore, age should be considered when weighing the risks and benefits of CCM treatment options. Other factors shown to increase the risk of epileptic seizures are CCM location and multiplicity.^9,25^ We categorized location based on the presence of a temporal CCM,^26^ specifically in cortical or superficial areas,^24,25^ but this was only associated in univariable analysis of epileptic seizure(s) after any presentation. The same was observed for CCM multiplicity, male sex, and largest CCM diameter, although CCM multiplicity was also associated in univariable analysis of seizure recurrence after a first unprovoked epileptic seizure due to CCM.

This study reports the final follow-up of a longstanding cohort study of patients with CCM, improving the precision of previously reported estimates by doubling the sample size and incorporating long-term follow-up.^9^ The increased statistical power enabled multivariable analyses, allowing for adjustments to potential confounders. This study had a population-based design restricted to incident cases of CCM to minimize selection bias related to treatment, symptoms, and other characteristics. Prospective follow-up reduced information bias, particularly recall bias, which is crucial when assessing seizure occurrence. Misclassification of a first-ever epileptic seizure and seizure recurrence after a first unprovoked epileptic seizure was minimized by thoroughly reviewing hospital and general practitioners' medical records to determine whether prepresentation epileptic seizure(s) had occurred. Moreover, rather than the often-used 1-year seizure freedom outcome, we analyzed 2-year seizure freedom which may be more relevant to clinical practice because withdrawal of ASM rarely occurs before a patient is 2-year seizure-free.^27^ Furthermore, we provided an additional 5-year seizure freedom outcome, which may be more meaningful for patients in the long term.

Some limitations should be considered in this study. First, there may have been informative censoring because patients were censored at CCM intervention, although treatment is generally related to the risk of events. Second, some events may have been missed because we relied on hospital and general practitioners' medical records and yearly patient questionnaires rather than scheduled study visits. Third, we may have misclassified some events as epileptic seizures and new FND that occur without radiologic evidence of ICH as well as migraine aura and Todd paresis can be difficult to distinguish.^2^ Fourth, we included ASM treatment after the first seizure in the multivariable analysis of seizure recurrence as a fixed binary covariate and did not account for the timing of ASM initiation, ASM adherence, or changes in type, number, or dosage of ASM. Such nonrandomized analysis of treatment effects must be interpreted with caution, which is why ASM use could not be included in other multivariable analyses. Instead, for the seizure freedom outcomes, we reported the number of ASM prescribed at the time of achieving seizure freedom as an exploratory variable.

This study enhances our understanding of seizure and epilepsy outcomes associated with CCM. During long-term follow-up, we found that the risk of a first-ever epileptic seizure is low, which should be reassuring for people diagnosed with CCM without seizures. Following a first unprovoked epileptic seizure due to CCM, the risk of recurrence is high, particularly if ASM treatment is not started after the first seizure, justifying diagnosis of CRE and initiation of ASM therapy after the first unprovoked epileptic seizure. Patients who meet the definition of CRE may achieve long-term seizure freedom with medical management, but for those who develop medically intractable seizures, selection for surgical treatment remains a case-by-case decision. Although some observational studies have shown promising reduction in seizure frequency before and after surgery, controlled studies confirming dramatic effects are lacking and existing surgical evidence regarding CRE is limited.^28^ Thus, to settle uncertainties and guide treatment decisions, a randomized controlled trial is needed, which has been shown to be feasible by a recent pilot study.^29^ Such a trial should focus on optimal surgical timing and approach, as well as ASM withdrawal after treatment.^30^ Until then, clinicians may use the findings from this study to inform patients with CCM about their risk of a first-ever epileptic seizure, the subsequent risk of seizure recurrence, and the chance of becoming seizure-free.

## Glossary

ASM: antiseizure medicine
CCM: cerebral cavernous malformation
CRE: CCM-related epilepsy
FND: focal neurologic deficit
HR: hazard ratio
ICH: intracranial hemorrhage
ILAE: International League Against Epilepsy
IQR: interquartile range
SAIVMs: Scottish Audit of Intracranial Vascular Malformations
